# Impact of educational interventions on the cultural competencies of nurses and students: a systematic review and meta-analysis

**DOI:** 10.1590/1980-220X-REEUSP-2025-0323en

**Published:** 2026-02-23

**Authors:** Sergio José Toribio Martínez, Paloma Echevarría Pérez, Francisca Sánchez Ayllón, José Luis Gómez Urquiza

**Affiliations:** 1 Universidad Católica San Antonio de Murcia, Programa de Doctorado en Ciencias de la Salud, Murcia, España.; 2 Universidad Católica San Antonio de Murcia, Facultad de Enfermería, Murcia, España.; 3 Universidad de Granada, Facultad de Ciencias de la Salud de Ceuta, Ceuta, España.

**Keywords:** Cultural Competence, Delivery of Health Care, Health Education, Systematic Review, Education, Nursing, Education, Competência Cultural, Atenção à Saúde, Educação em Saúde, Revisão Sistemática, Educação em Enfermagem, Educação

## Abstract

**Objective:**

To analyze the scientific literature on the effectiveness of educational interventions aimed at acquiring cultural competencies in nurses and nursing students.

**Method:**

Searches were conducted in the following databases PubMed, Lilacs, Esmerald, Scopus, and Scielo, focusing on the study of educational interventions related to cultural competencies. The PRISMA methodology was used, critical reading was performed with the CASPe tool, and random-effects meta-analyses were conducted with RevMan.

**Results:**

Thirteen studies were included, 11 of which were educational interventions. These significantly improved cultural competencies, especially in knowledge, communication skills, and self-awareness. Qualitatively, a positive impact was evidenced, and quantitatively, the effect on knowledge was statistically significant (P < 0.00001).

**Conclusion:**

The meta-analysis reveals a significantly positive effect, in a generalized way, of educational interventions on cultural competence in nurses and students. Continuous learning and innovative educational practices are necessary to maintain and reinforce knowledge. Further studies using both qualitative and quantitative methods are needed to improve both educational initiatives and cultural competence training.

## RESUMO

**Objetivo:**

Analisar a literatura científica sobre a eficácia das intervenções educativas orientadas à aquisição de competências culturais em enfermeiras e estudantes de enfermagem.

**Método:**

Foram realizadas buscas nas bases de dados PubMed, Lilacs, Esmerald, Scopus e Scielo, focadas no estudo de intervenções educativas relacionadas às competências culturais. Realizou-se com a metodologia PRISMA, a leitura crítica com a ferramenta CASPe e as meta-análises de efeitos aleatórios com o RevMan.

**Resultados:**

Foram incluídos 13 estudos, 11 foram intervenções educativas. Estas melhoraram significativamente as competências culturais, especialmente em conhecimentos, habilidades comunicativas e autoconsciência. Qualitativamente, evidenciou-se um impacto positivo e, quantitativamente, o efeito sobre o conhecimento foi estatisticamente significativo (P < 0,00001).

**Conclusão:**

A meta-análise revela um efeito significativamente positivo, de forma generalizada, das intervenções educativas na competência cultural em enfermeiras e estudantes. São necessários aprendizado contínuo e práticas educativas inovadoras para manter e reforçar conhecimentos. São necessários mais estudos que utilizem métodos qualitativos e quantitativos para a melhoria tanto em ações educativas quanto em capacitação em competência cultural.

## INTRODUCTION

Healthcare has undergone significant transformations in recent decades, especially in the global context^(1,2)^. These changes are the result of several factors, including demographic changes and increasing cultural diversity^(3)^.

Globally, healthcare has evolved to adapt to the changing needs of populations. The health systems of many countries have been under pressure due to the increase in chronic diseases, the aging population, and the growing expectations of patients^(4)^.

Globalization has led to greater mobility of people, resulting in the need for health systems to be more inclusive and adaptive^(5)^.

The World Health Organization (WHO) has identified the training of qualified personnel, equity, and quality as fundamental pillars for 21^st^-century health systems^(6)^. However, many countries still face challenges in ensuring equitable access to quality services^(7)^. The world is experiencing unprecedented demographic changes^(8)^.

Alongside these demographic changes, we are witnessing increasing cultural diversity in many regions. Migration, both voluntary and forced, has led to a mixing of cultures, religions, and traditions in many countries. The populations served by health systems are now more diverse than ever, which poses challenges and opportunities for health professionals^(9,10)^.

Cultural diversity in health goes beyond language; it requires understanding and respecting the beliefs, values, and practices of each patient. Cultural competence integrates behaviors, attitudes, and policies of the health system, applied by its professionals to ensure effective care in intercultural contexts^(11)^.

Cultural competencies have become an essential skill for nurses and nursing students, as they can influence the patient’s perception of care, adherence to treatment, and ultimately, health outcomes^(12)^.

A lack of cultural competence can have serious repercussions on the quality and effectiveness of care provided^(13,14,15)^. Cultural misunderstandings can arise from differences in beliefs, values, practices, and expectations between patients and health professionals.

Furthermore, a lack of cultural competence can lead to diagnostic errors, since certain symptoms or signs can be interpreted differently depending on the cultural context. Adverse outcomes related to a lack of cultural competence can have legal and ethical implications for professionals^(16)^.

It is important to emphasize the need for health training in this context, especially in the field of nursing, since value has been attributed to the importance of the patient-professional relationship and the growing diversity of the populations served, producing a shift towards a more holistic approach to training^(11)^.

These changes reflect a deeper understanding that health care is not just about diseases, but also about caring for people in their entirety.

The integration of cultural competence education into health training is essential to prepare professionals to work in a globalized and diverse environment^(16)^.

Training in cultural competence involves not only learning about different cultures, but also developing skills in self-awareness, empathy, communication and critical reflection^(10,13,14,15,16,17)^, in addition to being continuous and adaptive^(16)^.

There are hospitals which have even developed specific training in intercultural competence for professionals^(18)^. Therefore, the need for continuing education regarding the updating of cultural competences is truly necessary.

Educational interventions in cultural competence can transform health education, enabling nurses and students to relate effectively with diverse patients, improve quality and satisfaction, and reduce inequalities^(19)^. By strengthening communication, therapeutic adherence, early detection, and prevention are favored.

These educational interventions would result from the planned combination of learning experiences, based on theories and/or evidence, to update and improve knowledge, skills, and attitudes in professionals and students with the aim of providing culturally competent and equitable care^(20,21)^.

In addition, cultural competence will lead to better communication between nurses and patients, which can result in greater adherence to treatment, early detection of diseases, and more effective prevention.

Gallagher^(17)^ evaluated learning interventions to improve cultural competence in nurses and students. Increases in perceived competence (knowledge, skills, attitudes and self-efficacy) were found, nevertheless, it was highlighted that there is a lack of empirical evidence that this training translates into truly competent care or better health outcomes^(17)^.

Several studies show that improving this competence could be a strategy to reduce disparities in care for vulnerable groups. This research highlights the need to address cultural competence in nursing education and the importance of evaluating and improving educational interventions^(17,18)^.

Govere and Govere^(19)^ conducted another meta-analysis that addressed the growing need to provide culturally competent care and indicate that cultural competence training interventions have achieved a significant increase. In several of the studies they reviewed, a significant association was demonstrated between cultural competence training and an increase in patient satisfaction, concluding that cultural competence training is an effective intervention to better serve minority groups^(19)^.

Given the evidence on the positive impact of cultural competence in reducing care disparities and improving the satisfaction of vulnerable groups, educational interventions should be analyzed, so that these skills in nursing students and professionals can be further developed.

As previously justified, the topic is addressed in this research, we present in this article a systematic review whose objective is to analyze the effectiveness of educational interventions focused on cultural skills in nurses and nursing students.

## METHOD

### Study Design

The study methodology used was a systematic review following the guidelines established by the PRISMA (Preferred Reporting Items for Systematic Reviews and Meta-Analyses) statement with meta-analysis^(22)^. The study was registered on the PROSPERO platform with identification number “CRD420251185829”. The “ROBIS” (Risk Of Bias In Systematic Reviews) tool was used to assess bias in SRs. This tool evaluates four domains: eligibility criteria, study identification and selection, data collection, and synthesis.

### Inclusion Criteria

The studies considered for this review should meet the following criteria: publications from the last 10 years; related to educational interventions to improve cultural competencies in nurses and nursing students; reporting quantitative or qualitative measures of the effectiveness of educational interventions aimed at cultural competencies in both groups; and published both in English or Spanish.

### Exclusion Criteria

Opinion articles, editorials, and intervention studies conducted with healthcare professionals and students that did not provide independent data for nurses and nursing students were excluded.

### Search Strategy

The following databases were consulted: PubMed, Lilacs, Esmerald, Scopus, and Scielo, using the following search equations: (“Cultural competency” AND “Educational intervention” AND (“Health Workers” OR “Nurse” OR “Quality of Health Care”)) OR (educational interventions cultural competence); Cultural competency AND Educational intervention AND (Nurse OR Quality of Health Care); Cultural competency AND “Educational intervention” “Nurse”. The search strategy was not designed with specialized bibliometric support.

### Selection Process

**Figure 1:**
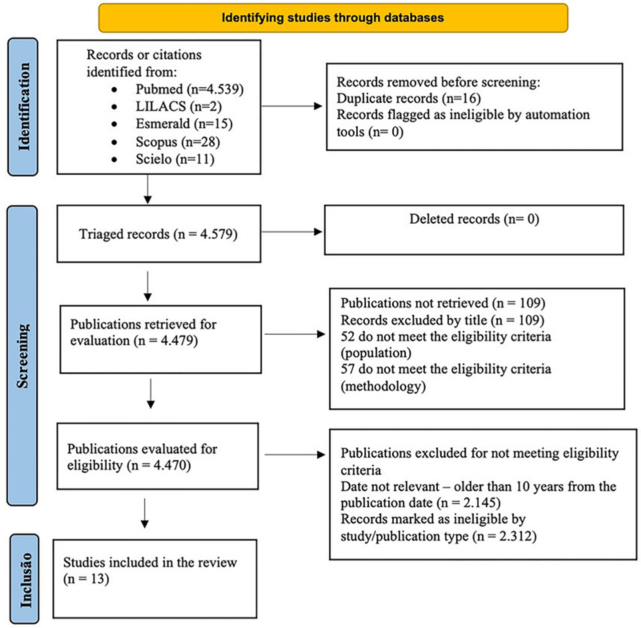
PRISMA flow diagram of the study selection process.

The initial search yielded 4,595 articles. Two researchers independently reviewed the titles and abstracts of the identified studies, assessing their eligibility according to previously established inclusion and exclusion criteria. After eliminating duplicates, potentially relevant studies underwent a fulltext reading. In cases of disagreement, a third researcher was consulted to reach a consensus. Once this process was completed, the final list of studies included in the review was compiled. For each study, the following data were systematically extracted: author, year of publication, country of origin, study objective, methodological design, sample size and characteristics, type of intervention, instruments used to assess cultural competence, and main results related to its effectiveness (the flowchart of the selection process is illustrated in Figure 1).

### Methodological Quality and Level of Evidence

Two reviewers from the group performed a critical reading and assessed the methodological quality of the selected articles by applying the checklists from the Critical Appraisal Skills Program Spanish (CASPe).

The level of evidence and the degree of recommendation of the included studies were assigned using the Sackett scale for clinical studies^(23)^. This tool considered aspects such as study design, sample selection, validity and reliability of the instruments used, and the adequacy of statistical analysis.

### Data Analysis aand Presentation of Results

The data were analyzed using a meta-analysis to combine the results of similar studies and obtain an overall estimate of the effect of the interventions using RevMan 5.4 software.

Three random-effects meta-analyses were performed, and the I index was calculated to measure heterogeneity^2^.

## RESULTS

In this systematic review, 13 studies were included (Figure 1), and most of the selected studies (n = 11) consisted of educational interventions aimed at improving cultural skills in health practice.

From the studies that provided specific demographic data, an average population of n = 646 participants was extracted. Among them, the number of men (M) was 87 (13.5%) and the number of women (W) was 462 (71.5%); in 15% of cases, the sex distribution was not reported. The average age (X̅) of the participants was 31.79 years.

In 8 of the studies, the data were compared with a similar control group (a total of 332 participants)^(24,25,26,27,28,29,31)^; in the remainder, no control group was applied, due to the methodology with which the studies were designed. These data can be seen in Table 1.

**Table 1:** General information, basic characteristics, results and educational actions used in the studies – Ceuta, CE, Spain, 2023.

Author, year (Country)	Type of design	Sample	Results	Level of evidence/Grade of recommendation	Educational actions used
Lin *et al.* 2015 (Taiwan)	Quasi-experimental longitudinal	N = 51 (M = 2 W = 49) Age X̅ = 20.4 Control group with 54 participants	Educational interventions improve cultural competencies in nursing students in the short term, but require continuous reinforcement and clinical practice to maintain their effectiveness.	3B	Formal conference with a question and answer (Q&A) session. Content focused on the definition of CC, theories, application, effects on quality of attention, and self-assessment/reflection.
Claramita *et al*. 2016 (Indonesia)	Quasi-experimental study with randomized controls	n = 14 (M = 4 W = 10) Age X̅ = NE Control group with 14 students who did not receive the guide.	Educational interventions improve culturally sensitive communication skills in nursing students, positively impacting patient satisfaction.	3B	Unique training using Gadjah Mada communication guides. Use of role-playing/playing to simulate culturally influenced clinical scenarios.
Chang *et al*. 2017 (Taiwan)	Randomized controlled trial	n = 65 (M = 50 W = 15) Age X̅ = 23.42 Control group with 55 participants	Interventions on *Facebook* increased cultural awareness among healthcare professionals, but did not significantly improve their knowledge or skills compared to the control group.	3B	Using *Facebook* as a platform to offer courses and educational materials. Content includes images, videos, text, and polls. Promotes social and personalized learning.
Young e Lu 2018 (Havaii)	Descriptive	n = 23 (M = 6 W = 17) Age X̅ = 29.2 Control group with 23 years old without intervention	The study demonstrates that the unique educational intervention improved cultural knowledge scores in nursing students while maintaining overall cultural competence, highlighting the importance of such interventions in health education.	C4	Intervention guided by a diagram and PowerPoint presentation. Didactic lectures, presentations, and discussions were used to provide an overview of cultural competence.
Cavalcante 2020 (Brazil)	Qualitative	n = 19 (M = 3 W = 16) Age X̅ = 39.6 There was no control group.	Educational interventions improved nurses’ critical cultural competencies, broadening cultural understanding and enhancing effective communication and healthcare delivery.	C4	Culture Circles with phases of thematic investigation, thematization, and problematization. Use of dynamics (“The Three Wishes,” “Tree of Life”). Visual techniques (Photovoice) and analysis (SWOT Matrix). Concrete product: idiomatic primer.
Lin e Hsu 2020 (Taiwan)	Blind randomized controlled trial	n = 50 (M = 0 W = 50) Age X̅ = 36.49 Control group with 50 nurses	The study revealed a positive effect of educational interventions on nurses’ cultural self-directed learning abilities, significantly improving this skill, which is relevant for strengthening cultural competencies in the context of healthcare.	3B	Intervention comprised of four units. Use of a film to analyze racial prejudices and stereotypes. Emphasis on communication and respect for diversity.
Mokel e Canty 2020 (USA)	Qualitative study	n = 24 (M = 0 W = 24) Age X̅ = 34.5 There was no control group.	Educational interventions based on the “Sunrise Enabler” improve the cultural competence of nursing students, promoting more culturally conscious awareness and benefiting both students and educators in developing practical skills and attitudes towards diversity.	C4	Use of Leininger’s Sunrise Enabler tool. Online journal for reflection and self-awareness. Online case discussion.
Kaiafas e Kennedy 2021 (USA)	PBE project with pre-and post-educational intervention	n = 36 (M = 7 W = 29) Age X̅ = 41.8 Previous control group of 36 professionals	The specific educational intervention significantly improved the knowledge, skills, openness, and support for the LGBTQ population among emergency room nurses, without impacting awareness of oppression, and is replicable in other departments.	3B	Educational intervention in two sessions. The AIM tool was used to evaluate the results (Knowledge, Awareness of Oppression, Openness).
Kula *et al*. 2021 (Israel)	Randomized controlled trial	n = 34 (M = 4 W = 30) Age X̅ = 33.71 Control group of 38 participants who did not receive the intervention.	Educational interventions improve cultural knowledge in nursing students, but not attitudes, suggesting the need for targeted programs and controlled trials to optimize training in cultural competence.	B2b	The program consisted of two 60-minute sessions. It included seven educational units on definitions of emergency, cultural challenges, introduction, attitudes, knowledge, and culturally competent skills.
Majda *et al.* 2021 (Poland)	Longitudinal	n = 130 (M = 4 W = 126) Age X̅ = 23.57 There was no control group.	Intercultural workshops enhance behavioral and cognitive aspects of cultural intelligence, and ongoing training is necessary for significant changes in skills and attitudes.	A1b	Intercultural communication workshops (20 hours: 10 practical sessions and 10 lectures). Use of active teaching strategies: simulation, role-playing, visualization, case studies, educational games, brainstorming.
Álvarez-San Martín *et al*. 2022 (Chile)	Descriptive cross-sectional quantitative	n = 97 (M = NE W = NE) Age X̅ = NE There was no control group.	Educational interventions improve students’ cultural competence, with greater development in cultural knowledge and skills than in cultural sensitivity.	C4	Assessment of existing competencies using the Cultural Competence Measurement Scale (EMCC-14). The incorporation of intercultural learning and individual/collective reflection into training is recommended.
Oikarainen *et al*. 2022 (Finland)	Non-randomized quasi-experimental	n = 49 (M = 5 W = 44) Age X̅ = 36,22 Control group with 62 participants receiving online tutoring but without education in cultural competence.	Mentor training significantly improves (p < 0.05) cultural competence in healthcare professionals, particularly in cultural interaction and safety, with a positive impact on the quality of care.	3B	Three-day teaching intervention. Use of online modules (flipped learning). Methods based on social constructivism: group discussions and simulation exercises.
Zeidani *et al*. 2023 (Iran)	Quasi-experimental	n = 54 (M = 2 W = 52) Age X̅ = 30,79 No control group	Communicative training significantly increased sensitivity and cultural competence in pediatric nurses, with sustained improvements after one month, supporting its integration into nursing education.	C4	Training in communication skills (*Communication skills training*).
Total	n = 646 (M = 87 W = 462) Age X̅ = 31,79				

The duration of the interventions varies significantly, from a single 2-3 hour session to programs lasting several weeks or a full semester. Studies indicate that short-term interventions, such as 2-3 hour workshops, can increase cultural knowledge, but are insufficient to achieve a deeper understanding or changes in attitudes and skills, since cultural competence is learned over time^(32)^. Table 1 shows the basic characteristics, results, methods used and educational actions employed in the studies.

### Results of the Meta-Analysis

#### Qualitative Improvement of Cultural Skills

**Figure 2:**
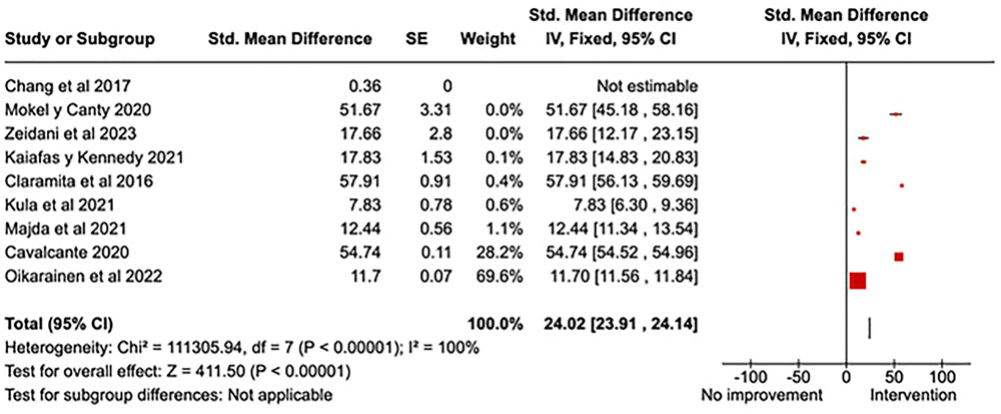
Improvement in qualitative cultural skills after educational interventions in healthcare professionals.

Out of the included studies, 9 (69.23%) were used to extract data on the qualitative improvement in attention as a function of interventions in cultural competencies (best practices, attitudes towards problems, professional perceptions regarding cultural diversity, and feedback with educators). All interventions that assessed the impact on these variables were effective in generating impact (confidence intervals that do not cross the zero value on the horizontal axis), as shown in Figure 2.

The diamond shape representing the combined total effect crosses the vertical axis at point 24.02, with a 95% Confidence Interval of [23.91, 24.14]. The results of the analyzed studies were highly heterogeneous (I2 = 100%). The test for the overall effect is highly significant (Z = 411.50, P < 0.00001).

### Quantitative Impact on Knowledge of Interventions in Cultural Skills

**Figure 3:**
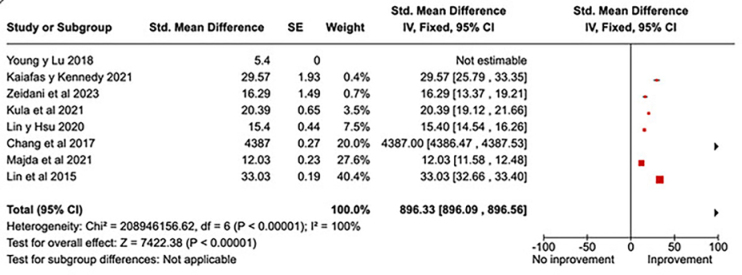
Quantitative improvement in knowledge about cultural competencies after educational interventions in healthcare professionals.

Eight studies were analyzed, and the overall result shows a considerably high standardized difference of means and a 95% confidence interval. The total effect of the intervention on knowledge of cultural competencies was statistically significant (P < 0.00001), as shown in Figure 3. The study by Lin et al.^(24)^ carries the most weight in relation to this variable.

#### Impact on Professional Confidence

**Figure 4:**
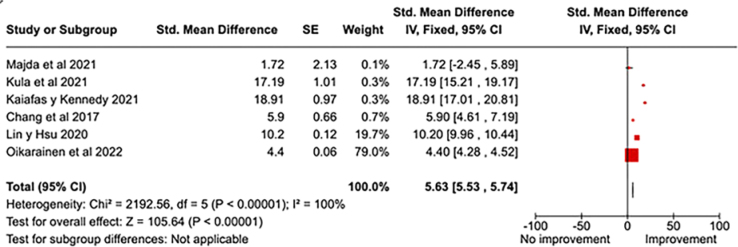
Impact of factors related to cultural competencies that influence the improvement of professional confidence.

Six studies were evaluated to determine the impact of factors related to cultural competencies on improving professional confidence. The overall analysis reflects a significant standardized difference in means, with a 95% confidence interval, highlighting a positive impact on confidence. The combined effect of these interventions proved to be statistically significant with a P-value < 0.00001. These results are presented in Figure 4. The study with the greatest impact on the analysis was that of Oikarainen et al.^(31)^, which provides the greatest weight in relation to this specific variable.

## DISCUSSION

This analysis delves into the impact of educational interventions on the development of cultural competence in the health field, revealing a predominant emphasis oriented towards the development of intercultural understanding and skills applied to the clinical context, as established by Lin et al.^(24)^; Claramita et al.^(25)^ and other authors^(26,27,28,29,30,31,32,35)^. Most of the analyzed studies showed that the interventions have significantly influenced the improvement of the cultural competence of students and nurses^(36)^.

Multiple studies agree that cultural competence is a multidimensional construct that encompasses knowledge, sensitivity/awareness, skills and, in some models, desire and cultural encounter. Educational activities seek to develop these sub-areas in an integral and coordinated manner^(37,38)^. As indicated in Table 1, it is possible to appreciate the various educational actions used to foster deeper and more transformative learning, recognizing that a unidimensional approach is insufficient to address the complexity of this construct^(39,40)^.

Educational interventions include lectures, question and answer sessions, and debates to cover definitions, importance, theories, and the cultural impact on health care. However, it has been pointed out that didactic lectures alone may not be sufficient to develop all dimensions of cultural competence, particularly those related to cultural sensitivity and practical application skills in real clinical contexts^(36,37)^.

The use of videos and films, followed by interactive discussions, represents an effective educational strategy that helps to understand cultural diversity, eliminate stereotypes and prejudices, and foster reflection on one’s own values and the impact of cultural backgrounds. This methodology allows participants to visualize culturally complex situations and develop greater awareness of intercultural dynamics in the context of health care^(40)^.

Reflective writing exercises on one’s own cultural heritage, often combined with drawings and discussion, facilitate self-exploration and understanding of how personal culture influences health decisions. It is noteworthy that reflection is a crucial part of designing activities for cultural competence, as it allows health professionals to recognize their own cultural biases and develop greater self-awareness before working with diverse populations^(37)^.

In the development of these interventions, recognized models such as Giger and Davidhizar’s, Campinha-Bacote’s Cultural Competence Process, and Leininger’s Cultural Care (Sunrise Enabler) Theory are used. These models provide solid theoretical frameworks for the evaluation and design of culturally sensitive interventions, offering conceptual structures that guide both the implementation and evaluation of educational programs^(37)^.

The emphasis on the “clinical context” suggests that the most effective interventions are not only theoretical, but they incorporate practical cases, simulations, or scenarios in which students and professionals need to apply their knowledge of cultural diversity in real health care situations. Among these strategies, interactive case studies stand out, in which cultural dilemmas are presented and the best ways to address them in a clinical setting are discussed; Simulations with standardized patients, which train participants to interact with “patients” from different cultural contexts and to manage delicate situations; and clinical rotations or cultural immersion experiences, the latter being one of the most powerful interventions included in the scientific literature^(39,40)^.

Case studies and simulations allow students to analyze complex situations, practice skills in a safe environment, and apply knowledge to realistic scenarios. Structured role-playing, in particular, can help to experience inequality and reflect on biases, providing a transformative learning experience that goes beyond theoretical knowledge, towards a lived understanding of cultural dynamics in health care^(40)^.

The predominance of women in the studied population (71.5%) and an average age of the subjects in the initial or intermediate phases of their professional trajectory suggest that the promotion of cultural competencies is being prioritized in fundamental formative stages, as suggested by Álvarez-San Martín et al.^(34)^. This approach can be strategic, considering that early socialization in cultural competencies can have a lasting effect on professional practice, providing a solid foundation for its continuous development.

The use of *Facebook* as an educational tool, according to Chang et al.^(26)^, and the use of Culture Circles by Cavalcante^(33)^ reflect a trend towards pedagogical innovation in the teaching of cultural competence. Giroux and Moreau^(41)^, in their article, indicate that social networks play a significant role in the development of academic activities. In the case of *Facebook*, its effectiveness lies in its use as a platform for online discussions, sharing multimedia resources and fostering critical reflection on cultural cases outside the classroom, promoting continuous interaction and collaborative learning^(42,43)^.

As for Culture Circles, this approach requires a structured method that involves dialogue, group reflection and collective construction of knowledge on cultural themes. It is an approach that values the lived experiences of the participants and promotes empathy and mutual understanding through guided discussion.

The integration of these platforms in the educational field presents relevant considerations for the theoretical frameworks of learning and for the instructional design of academic programs. In particular, there is a need to conceptualize and enable virtual spaces that facilitate the formation of communities of practice and collaborative learning among students, recognizing the pedagogical potential of these digital environments to enrich the educational experience and promote the social construction of knowledge^(42,43)^.

Nevertheless, a significant methodological limitation is identified: the scarce use of control groups, present in only 8 of the 13 studies analyzed. This lack suggests the need for more robust methodological structures in future research to conclusively assess the effectiveness of these interventions^(36,44)^.

The diversity in assessment methodologies and tools, along with the variability of interventions, underlines the complexity of standardizing cultural competence measurements and interventions, as established by other authors such as Kaiafas and Kennedy^(29)^ and Kula et al.^(30)^. This heterogeneity poses significant challenges for standardization and reliable comparison of findings between different studies^(38,45)^. Despite these differences, the overall trend of improved confidence and knowledge about cultural competencies among participants points to a positive effect of the interventions, a statement with which Majda et al.^(32)^ and Zeidani et al.^(35)^ agree.

The focus on the training of nursing students reflects a vanguard in this educational area, but also marks the importance of expanding the scope of these studies to other health specialties, in order to obtain a more holistic perspective on the influence and impact of cultural competencies in the health field, as established by Oikarainen et al.^(31)(40,45)^.

In the meta-analysis, it is observed that all individual studies show a positive effect of the interventions (Figure 2). The combined effect size indicates a significant improvement in cultural competencies as a result of the interventions. The Z-value of 4.11 with a p-value less than 0.00001 indicates high statistical significance of the overall result; in other words, it is very unlikely that this result is due to chance. Although there is considerable heterogeneity among the studies – possibly due to differences in interventions, in the populations studied, or in how cultural competencies were measured – the consistency in the direction of the effect reinforces the idea that educational interventions have a positive impact on improving cultural competencies in health professionals, which suggests the relevance of incorporating and enhancing cultural learning strategies in the continuing education of health professionals^(36)^.

Truong et al.^(46)^, in their review of reviews, indicate that there is some evidence that interventions to improve cultural competence can improve patient/client health outcomes, although evidence on patient outcomes remains limited and heterogeneous^(44)^.

It has been shown that educational interventions have a substantial effect on knowledge about cultural competencies among health professionals. The study by Lin et al.^(24)^ stands out within the analysis, suggesting that the nature or quality of this particular intervention is especially effective^(24,36)^.

The robustness of the results reinforces the researcher’s premise that a directed and well-structured education can be a pillar in improving the quality of health services, which agrees with what has been proposed by several authors who have carried out similar reviews in the past^(17,18,19,36,44)^.

These findings show that educational interventions increase understanding and develop the ability of professionals to act sensitively in the face of cultural diversity. Similarly, the results highlight the relevance of considering the methodological specificity of each intervention, since certain training strategies demonstrate greater effectiveness than others in developing this fundamental competence for health care in globalized contexts(38,40,45).

In the analysis of the factors that influence professional confidence, it is observed that cultural competence interventions play a significant role, as reflected in the study by Oikarainen et al.^(31)^, which emerges as the most influential in this aspect, and in that of Osmancevic et al.^(36)^.

The investigation encompasses a variety of studies that demonstrate the positive impact of educational interventions on cultural competencies. The improvement in confidence and cultural knowledge among health professionals indicates that such interventions are crucial. Furthermore, the focus on training nursing students and the innovative use of tools such as *Facebook* suggest an adaptation of teaching methods to current trends and emerging needs in the health sector^(40,42,43)^.

Nevertheless, this study presents several limitations that should be considered when interpreting the findings. Among the study’s limitations, the variability in sample sizes and demographic characteristics stands out, which implies caution in generalizing the results; in addition, although all studies indicate an improvement, the high heterogeneity observed means that the magnitude of this improvement varies considerably.

One of the most evident limitations is the absence of control groups in a considerable proportion of the studies, which makes it difficult to objectively compare the results and to causally attribute the improvements to specific interventions. Furthermore, the heterogeneity observed in the methodologies and assessment tools used poses challenges to standardizing and reliably comparing the results, as warned by Kaiafas and Kennedy^(29)^ and Kula et al.^(30)^. Variability in sample sizes and differences in the demographic characteristics of participants also limit the ability to generalize the findings to the general population. Finally, although all individual studies point to an improvement, the high heterogeneity in the observed effects suggests that the magnitude of this improvement varies considerably, which requires caution when inferring the magnitude of the overall impact.

For future research, it would be beneficial to structure studies with well-defined control groups and homogeneous methodologies in order to strengthen the validity of the conclusions. Similarly, it would be pertinent to explore the impact of these educational interventions in a wider range of health disciplines and at different professional stages, not limited to nursing alone. Likewise, it would be valuable to investigate the long-term permanence of acquired cultural competencies and how they apply in daily practice. Finally, it is recommended to conduct large-scale randomized controlled trials for a more precise evaluation of the effectiveness of cultural competence interventions and the contextualized adaptation of educational programs to different patient populations.

## CONCLUSION

Educational interventions in cultural competence are fundamental for the improvement of the quality of healthcare, increasing patient satisfaction and the intercultural communication skills of professionals. Cultural competence is a dynamic process that requires continuous training, critical self-reflection, and cultural humility to overcome personal biases.

Although significant challenges exist (linguistic barriers, cultural differences in conceptions of health, precarious working conditions), overcoming them is essential through the development of intercultural communication skills and the appreciation of local community knowledge.

Problems persist in evaluating attitudinal changes; short interventions do not consolidate the affective component, and the functionalist approach predominates over critical perspectives, while precarious employment, political influences, and lack of resources interrupt educational continuity.

In summary, the evidence suggests benefits, however, more robust studies are needed to clarify which pedagogical components generate the greatest effects and in which contexts. Standardization of metrics beyond self-assessment, incorporating clinical outcomes, is a priority. Randomized trials and longitudinal designs with ≥6–12 months of follow-up are lacking to estimate the effect and, therefore, the response. Analyses of moderators and mediators (professional profile, care environment, workload, organizational support) are needed to explain heterogeneity. It is urgent to incorporate underrepresented contexts (primary care, emergency rooms, ICUs; lower-middle-income countries) and mixed methods that link mechanisms to outcomes. Finally, there are almost no economic evaluations or studies on the impact of digital interventions.

## Data Availability

The entire dataset supporting the results of this study is available upon request to the corresponding author.
