# Supplementary material for: Impact of educational interventions on the cultural competencies of nurses and students: a systematic review and meta-analysis
Source: Rev Esc Enferm USP. 2026 Feb 23;60:e20250323. doi: 10.1590/1980-220X-REEUSP-2025-0323en (PMC12928726; doi:10.1590/1980-220X-REEUSP-2025-0323en)
Supplement: Translation

# Translation: Impacto de intervenciones educativas en competencias culturales de enfermeras y estudiantes: revisión sistemática y meta-análisis

**DOI:** 10.1590/1980-220X-REEUSP-2025-0323es

**Authors:** Sergio José Toribio Martínez, Paloma Echevarría Pérez, Francisca Sánchez Ayllón, José Luis Gómez Urquiza

**Affiliations:** 1 Universidad Católica San Antonio de Murcia, Programa de Doctorado en Ciencias de la Salud, Murcia, España.; 2 Universidad Católica San Antonio de Murcia, Facultad de Enfermería, Murcia, España.; 3 Universidad de Granada, Facultad de Ciencias de la Salud de Ceuta, Ceuta, España.

**Keywords:** Competencia Cultural, Atención a la Salud, Educación en Salud, Revisión Sistemática, Educación en Enfermería, Educación

## Abstract

**Objetivo:**

Analizar la literartura científica sobre la eficacia de las intervenciones educativas orientadas a la adquisición de competencias culturales en enfermeras y estudiantes de enfermería.

**Método:**

Se realizaron búsquedas en las bases de datos PubMed, Lilacs, Esmerald, Scopus, y Scielo, enfocadas al estudio de intervenciones educativas relacionadas con las competencias culturales. Se realizó con metodología PRISMA, la lectura crítica con la herramienta CASPe y los metaanálisis de efectos aleatorios con RevMan.

**Resultados:**

Se incluyeron 13 estudios, 11 fueron intervenciones educativas. Estas mejoraron significativamente las competencias culturales, especialmente en conocimientos, habilidades comunicativas y autoconciencia. Cualitativamente, se evidenció un impacto positivo, y cuantitativamente el efecto sobre el conocimiento fue estadísticamente significativo (P < 0.00001).

**Conclusión:**

El metaanálisis revela un efecto significativamente positivo de forma generalizada de las intervenciones educativas en la competencia cultural en enfermeras y estudiantes. Es necesario el aprendizaje continuo y prácticas educativas innovadoras para mantener y reforzar conocimientos. Se necesitan más estudios que utilicen métodos cualitativos y cuantitativos para la mejora tanto en acciones educativas como en capacitación en competencia cultural.

## INTRODUCCIÓN

La atención sanitaria ha experimentado transformaciones significativas en las últimas décadas, especialmente en el contexto global^(1,2)^. Estos cambios producidos son el resultado de diversos factores, incluyendo cambios demográficos y una diversidad cultural creciente^(3)^.

A nivel mundial, la atención sanitaria ha evolucionado para adaptarse a las necesidades cambiantes de las poblaciones. Los sistemas de salud de muchos países han experimentado presiones debido al aumento de enfermedades crónicas, el envejecimiento de la población y las crecientes expectativas de los pacientes^(4)^.

La globalización ha llevado a una mayor movilidad de las personas, dando como resultado la necesidad de que sistemas de salud sean más inclusivos y adaptativos^(5)^.

La Organización Mundial de la Salud (OMS) ha identificado la formación de personal cualificado, la equidad y la calidad como pilares fundamentales para los sistemas de salud del siglo XXI^(6)^. Sin embargo, muchos países aún presentan desafíos para garantizar el acceso equitativo a servicios de calidad^(7)^. El mundo está experimentando cambios demográficos sin precedentes^(8)^.

Paralelamente a estos cambios demográficos, estamos presenciando una diversidad cultural creciente en muchas regiones. La migración, tanto voluntaria como forzada, ha llevado a una mezcla de culturas, religiones y tradiciones en muchos países. Las poblaciones atendidas en los sistemas de salud son ahora más diversas que nunca, lo que plantea desafíos y oportunidades para los profesionales sanitarios^(9,10)^.

La diversidad cultural en salud va más allá del idioma, exige comprender y respetar las creencias, valores y prácticas de cada paciente. La competencia cultural integra comportamientos, actitudes y políticas del sistema sanitario, aplicadas por sus profesionales para garantizar una atención eficaz en contextos interculturales^(11)^.

Las competencias culturales se han convertido en una habilidad esencial para enfermeras y estudiantes de enfermería, ya que pueden influir en la percepción del paciente sobre la atención, la adherencia al tratamiento y, en última instancia, los resultados de salud^(12)^.

La falta de competencia cultural puede tener graves repercusiones en la calidad y eficacia de la atención proporcionada^(13,14,15)^. Los malentendidos culturales pueden surgir de diferencias en las creencias, valores, prácticas y expectativas entre pacientes y profesionales de salud.

Además, la falta de competencia cultural puede llevar a errores diagnósticos, ya que ciertos síntomas o signos pueden interpretarse de manera diferente según el contexto cultural. Los resultados adversos relacionados con la falta de competencia cultural pueden tener implicaciones legales y éticas para los profesionales^(16)^.

Es importante resaltar la necesidad de formación sanitaria en este contexto sobre todo en el área de enfermería, ya que se ha dado valor a la importancia de la relación paciente-profesional y a la creciente diversidad de las poblaciones atendidas, produciendo un cambio hacia un enfoque más holístico de la formación^(11)^.

Estos cambios reflejan una comprensión más profunda de que la atención sanitaria, no solo se trata enfermedades, sino también de cuidar a las personas en su totalidad.

La integración de la enseñanza de competencias culturales en la formación sanitaria es esencial para preparar a los profesionales para trabajar en un entorno globalizado y diverso^(16)^.

La formación en competencia cultural no solo implica aprender sobre diferentes culturas, sino también desarrollar habilidades de autoconciencia, empatía, comunicación y reflexión crítica^(10,13,14,15,16,17)^, además de ser continua y adaptativa^(16)^.

Existen hospitales en los que se llegan a desarrollar entrenamientos específicos en competencia intercultural para profesionales^(18)^. Por ello la necesidad de formación continuada referente a la actualización de las competencias culturales es realmente necesaria.

Las intervenciones educativas en competencia cultural pueden transformar la formación sanitária, capacitando a enfermeras y estudiantes para relacionarse eficazmente con pacientes diversos, mejoran la calidad y la satisfacción y reducen desigualdades^(19)^. Al fortalecer la comunicación, se favorece la adherencia terapéutica, la detección precoz y la prevención.

Estas intervenciones educativas resultarían de la combinación planificada de experiencias de aprendizaje, basadas en teorías y/o evidencia, para actualizar y mejorar conocimientos, habilidades y actitudes en profesionales y estudiantes con el fin, de proporcionar una atención culturalmente competente y equitativa^(20,21)^.

Además, la competencia cultural llevará a una mejor comunicación entre las enfermeras y pacientes, lo que puede resultar en una mayor adherencia al tratamiento, una detección temprana de enfermedades y una prevención más efectiva.

Gallagher^(17)^ evaluó intervenciones de aprendizaje para mejorar la competencia cultural en enfermeras y estudiantes. Halló aumentos en la competencia percibida (conocimientos, habilidades, actitudes y autoeficacia), pero advirtió que falta evidencia empírica para que esta formación se traduzca en una atención realmente competente o en mejores resultados de salud^(17)^.

Varios estudios muestran que mejorar esta competencia podría ser una estrategia para reducir las disparidades en la atención de grupos vulnerables. Esta investigación subraya la necesidad de abordar la competencia cultural en la formación de enfermería y la importancia de evaluar y mejorar las intervenciones educativas^(17,18)^.

Govere y Govere^(19)^ llevaron a cabo otro metaanálisis que abordó la creciente necesidad de ofrecer atención culturalmente competente, y señalan que las intervenciones de formación en competencia cultural lograron un aumento significativo. En varios de los estudios que revisaron se demostró una asociación significativa entre la formación en competencia cultural y un aumento en la satisfacción del paciente, concluyendo que la formación en competencia cultural es una intervención efectiva para atender mejor a grupos minoritarios^(19)^.

Dada la evidencia sobre el impacto positivo de la competencia cultural en la reducción de disparidades asistenciales y la mejora de la satisfacción de grupos vulnerables, resulta necesario analizar las intervenciones educativas dirigidas al desarrollo de estas competencias en estudiantes y profesionales de enfermería.

Una vez justificada la importancia de profundizar en el tema tratado en esta investigación, planteamos en el siguiente artículo una revisión sistemática cuyo objetivo es analizar la eficacia de las intervenciones educativas orientada a las competencias culturales en enfermeras y estudiantes de enfermería.

## MÉTODO

### Diseño Del Estudio

La metodología de estudio utilizada fue una revisión sistemática siguiendo las directrices marcadas por la declaración PRISMA (Preferred Reporting Items for Systematic Reviews and Meta-Analyses) con metaanálisis^(22)^. El estudio se registró en la plataforma PROSPERO con nº de identificación “CRD420251185829”. Se utilizó la herramienta “ROBIS” (Risk Of Bias In Systematic Reviews) para evaluar el sesgo en RS. Esta herramienta evalúa cuatro dominios: criterios de elegibilidad, identificación y selección de estudios, recopilación de datos y síntesis.

### Criterios de Inclusión

Los estudios considerados para esta revisión debían cumplir con los siguientes criterios: publicaciones realizadas en los últimos 10 años; relacionados con intervenciones educativas para la mejora de la competencias culturales en enfermeras y estudiantes de enfermería; que reporten medidas cuantitativas o cualitativas de la eficacia de las intervenciones educativas orientadas a las competencias culturales en ambos grupos y publicados en idiomas inglés o español.

### Criterios de Exclusión

Se excluyeron artículos de opinión, editoriales y aquellos estudios de intervención realizados sobre profesionales sanitarios y estudiantes que no daban datos independientes para enfermeras y estudiantes de enfermería.

### Estrategia de Búsqueda

Se consultaron las siguientes bases de datos: PubMed, Lilacs, Esmerald, Scopus y Scielo, siendo las ecuaciones de búsqueda: (“Cultural competency” AND “Educational intervention” AND (“Health Workers” OR “Nurse” OR “Quality of Health Care”)) OR (educational interventions cultural competence); Cultural competency AND Educational intervention AND (Nurse OR Quality of Health Care; Cultural competency AND “Educational intervention” “Nurse”. La estrategia de búsqueda no fue diseñada con apoyo bibliométrico especializado.

### Proceso de Selección

La búsqueda inicial obtuvo 4595 artículos. Dos investigadores revisaron de forma independiente los títulos y resúmenes de los estudios identificados, evaluando su elegibilidad según los criterios de inclusión y exclusión previamente establecidos. Tras eliminar los duplicados, los estudios potencialmente relevantes fueron sometidos a una lectura completa. En los casos de desacuerdo, se recurrió a un tercer investigador para alcanzar consenso. Finalizado este proceso, se elaboró la lista definitiva de estudios incluidos en la revisión. Para cada uno de ellos, se extrajeron de forma sistemática los siguientes datos: autor, año de publicación, país de origen, objetivo del estudio, diseño metodológico, tamaño y características de la muestra, tipo de intervención, instrumentos utilizados para evaluar la competencia cultural y principales resultados relacionados con su efectividad (el diagrama de flujo del proceso de selección se ilustra en la Figura 1).

**Figura 1:** Diagrama de flujo PRISMA del proceso de selección de estudios.

### Calidad Metodológica y Nivel de Evidencia

Dos evaluadores del grupo llevaron a cabo una lectura crítica y evaluaron la calidad metodológica de los artículos seleccionados aplicando los checklist de Critial Appraisal Skills Program Spanish (CASPe).

El nivel de evidencia y grado de recomendación de los estudios incluidos se asignó utilizando la escala de Sackett para estudios clínicos^(23)^. Esta herramienta consideró aspectos como el diseño del estudio, la selección de la muestra, la validez y fiabilidad de los instrumentos utilizados, y la adecuación del análisis estadístico.

### Análisis de Datos y Presentación de Resultados

Los datos se analizaron por medio de un metaanálisis para combinar los resultados de estudios similares y obtener una estimación global del efecto de las intervenciones utilizando el software RevMan 5.4.

Se realizaron 3 metaanálisis de efectos aleatorios y para medir la heterogeneidad se calculó el índice I^2^.

## RESULTADOS

En la presente revisión sistemática se incluyeron 13 estudios (Figura 1), la mayoría de los estudios seleccionados (n = 11) fueron intervenciones educativas dirigidas a mejorar las habilidades culturales en la práctica sanitaria.

De los estudios que proporcionaron datos demográficos específicos, se extrajo una población media de n = 646 participantes. Entre ellos, la cantidad de hombres (H) fue de 87 (13,5%) y la de mujeres (M) 462 (71,5%), y en 15% de los casos no se reportó la distribución por sexo. La media de edad (X̅) de los participantes fue de 31,79 años.

En 8 de los estudios los datos fueron comparados con un grupo control similar (un total de 332 participantes)^(24,25,26,27,28,29,30,31)^, en el resto no se aplicó grupo control, debido a la metodología con la que se plantearon los estudios. Estos datos se pueden evidenciar en la Tabla 1.

**Tabla 1:** Información general, características básicas, resultados y acciones educativas utilizadas en los estudios – Ceuta, CE, España, 2023.

Autor, año (País)	Tipo de diseño	Muestra	Resultados	Nivel de evidencia/Grado recomendación	Acciones educativas utilizadas
Lin et al. 2015 (Taiwán)	Cuasiexperimental longitudinal	n = 51 (H = 2 M = 49) Edad X̅ = 20,4 Grupo control con 54 participantes	Las intervenciones educativas mejoran las competencias culturales en estudiantes de enfermería a corto plazo, pero requieren refuerzo continuo y práctica clínica para mantener su efectividad.	3B	Conferencia formal con sesión de preguntas y respuestas (Q&A). Contenido centrado en la definición de CC, teorías, aplicación, efectos en la calidad de la atención y autoevaluación/reflexión.
Claramita et al. 2016 (Indonesia)	Estudio cuasiexperimental control aleatorio	n = 14 (H = 4 M = 10) Edad X̅ = NE Grupo control con 14 estudiantes que no recibieron la guía	Las intervenciones educativas mejoran habilidades de comunicación culturalmente sensibles en estudiantes de enfermería, afectando positivamente la satisfacción del paciente.	3B	Entrenamiento único utilizando Guías de comunicación Gadjah Mada. Uso de dramatizaciones/juegos de roles para simular escenarios clínicos influenciados culturalmente.
Chang et al. 2017 (Taiwán)	Ensayo controlado aleatorio	n = 65 (H = 50 M = 15) Edad X̅ = 23,42 Grupo control con 55 participantes	Las intervenciones en Facebook aumentaron la conciencia cultural en profesionales sanitarios, pero no mejoraron significativamente los conocimientos o habilidades comparado con el grupo control.	3B	Uso de Facebook como plataforma de entrega para cursos y materiales educativos. Contenido con imágenes, vídeos, texto y encuestas.Promueve el aprendizaje social y personalizado.
Young y Lu 2018 (Hawai)	Descriptivo	n = 23 (H = 6 M = 17) Edad X̅ = 29,2 Grupo control con 23 sin intervención	El estudio demuestra que la intervención educativa única mejoró la puntuación de conocimiento cultural en estudiantes de enfermería, manteniendo la competencia cultural general, resaltando la importancia de tales intervenciones en la formación sanitaria.	C4	Intervención guiada por un esquema y presentación en Power Point. Se utilizó conferencia didáctica, presentación y discusión para proporcionar una visión general de la competencia cultural.
Cavalcante 2020 (Brasil)	Cualitativo	n = 19 (H = 3 M = 16) Edad X̅ = 39,6 No hubo grupo control	Las intervenciones educativas mejoraron las competencias culturales críticas de enfermeros, ampliando la comprensión cultural y mejorando la comunicación efectiva y el cuidado de la salud.	C4	Cículos de Cultura con fases de investigación temática, tematización y problematización. Uso de dinámicas (“Los tres deseos”, “Árbol de la Vida”). Técnicas visuales (Photovoice) y de anállisis (Matriz Fofa/Swot). Producto concreto: Carilla idiomática.
Lin y Hsu 2020 (Taiwán)	Ensayo controlado aleatorizado a ciegas	n = 50 (H = 0 M = 50) Edad X̅ = 36,49 Grupo control con 50 enfermeras	El estudio reveló un efecto positivo de las intervenciones educativas en la capacidad autodidacta cultural de las enfermeras, mejorando esta habilidad de forma significativa, lo cual es relevante para fortalecer competencias culturales en el ámbito sanitario.	3B	Intervención compuesta por cuatro unidades. Uso de una película para analizar prejuicios y estereotipos raciales. Énfasis en la comunicación y respeto a la diversidad.
Mokel y Canty 2020 (USA)	Estudio cualitativo	n = 24 (H = 0 M = 24) Edad X̅ = 34,5 No hubo grupo control	Las intervenciones educativas basadas en el “Sunrise Enabler” mejoran la competencia cultural de estudiantes de enfermería, promoviendo una atención más consciente culturalmente y beneficiando tanto a estudiantes como educadores en el desarrollo de habilidades prácticas y actitudes hacia la diversidad.	C4	Uso de la Herramienta Facilitadora del Amanecer de Leininger (Sunrise Enabler). Diario en línea para reflexión y autoconciencia. Discusión en línea de casos.
Kaiafas y Kennedy 2021 (USA)	Proyecto de PBE con intervención pre y post-educacional	n = 36 (H = 7 M = 29) Edad X̅ = 41,8 Grupo control previo de 36 profesionales	La intervención educativa específica mejoró significativamente el conocimiento, habilidades, apertura y apoyo hacia la población LGBTQ en enfermeras de emergencia, sin impactar la conciencia de opresión, siendo replicable en otros departamentos.	3B	Intervención educativa en dos sesiones. Se utilizó la herramienta AIM para evaluar los resultados (Conocimiento, Conciencia de la Opresión, Apertura).
Kula et al. 2021 (Israel)	Ensayo controlado aleatorio	n = 34 (H = 4 M = 30) Edad X̅ = 33,71 Grupo control de 38 participantes que no recibieron la intervención	Las intervenciones educativas mejoran el conocimiento cultural en estudiantes de enfermería pero no las actitudes, sugiriendo la necesidad de programas localizados y ensayos controlados para optimizar la formación en competencia cultural.	B2b	Programa que consistió en dos sesiones de 60 minutos. Incluyó siete unidades educativas sobre definiciones de emergencia, desafíos culturales, introducción, actitudes, conocimientos y habilidades culturalmente competentes.
Majda et al. 2021 (Polonia)	Longitudinal	n = 130 (H = 4 M = 126) Edad X̅ = 23,57 No hubo grupo control	Los talleres interculturales potencian aspectos comportamentales y cognitivos de la inteligencia cultural, necesitando formación continuada para cambios significativos en competencias y actitudes.	A1b	Talleres de comunicación intercultural (20 horas: 10 prácticas y 10 conferencias). Uso de estrategias de enseñanza activa: simulación, juego de roles, visualización, casos, juegos didáctivos, lluvia de ideas.
Álvarez-San Martín et al. 2022 (Chile)	Cuantitativo transversal descriptivo	n = 97 (H = NE M = NE) Edad X̅ = NE No hubo grupo control	Las intervenciones educativas mejoran la competencia cultural estudiantil, con mayor desarrollo en conocimiento y habilidades culturales que en sensibilidad cultural.	C4	Evaluación de competencias existentes mediante la Escala de Medición de Competencia Cultural (EMCC-14). Se recomienda la incorporación de aprendizaje intercultural y reflexión individual/colectiva en la formación.
Oikarainen et al. 2022 (Finlandia)	Cuasiexperimental no aleatorizado	n = 49 (H = 5 M = 44) Edad X̅ = 36,22 Grupo control con 62 participantes con tutoría online sin educación en competencia cultural	La formación de mentores mejora significativamente (p < 0,05) la competencia cultural en profesionales sanitarios, destacando en interacción cultural y seguridad, con impacto positivo en la calidad asistencial.	3B	Intervención de tres días de enseñanza. Uso de módulos en línea (flipped learnig). Métodos basados en constructivismo social: discusiones grupales y ejerccios de simulación.
Zeidani et al. 2023 (Irán)	Cuasiexperimental	n = 54 (H = 2 M = 52) Edad X̅ = 30,79 No grupo control	La formación comunicativa aumentó significativamente la sensibilidad y competencia cultural en enfermeros pediátricos, con mejoras sostenidas al mes, respaldando su integración en la educación enfermera.	C4	Capacitación en habilidades de comunicación (Communication skills training).
Total	n = 646(H = 87 M = 462) Edad X̅ = 31,79				

La duración de las intervenciones varía significativamente, desde una única sesión de 2-3 horas hasta programas de varias semanas o un semestre completo. Los estudios indican que las intervenciones a corto plazo, como talleres de 2-3 horas, pueden aumentar el conocimiento cultural pero son insuficientes para lograr una comprensión más profunda o cambios en actitudes y habilidades, ya que la competencia cultural se aprende con el tiempo^(32)^. En la Tabla 1 se indican las características básicas, resultados, métodos utilizados y las acciones educativas utilizadas en los estudios.

### Resultados del Meta-Análisis

#### Mejora Cualitativa de Competencias Culturales

De los estudios incluidos, 9 (69,23%) se usaron para extraer datos sobre la mejora cualitativa de la atención en función a las intervenciones de competencias culturales (mejores prácticas, actitudes ante problemas, percepciones profesionales hacia la diversidad cultural y feedback con educadores), todas las intervenciones que evaluaron el impacto sobre estas variables fueron efectivas en crear un impacto (intervalos de confianza que no cruzan el valor cero en el eje horizontal), tal y como se muestra en la Figura 2.

**Figura 2:** Mejora en competencias culturales cualitativas tras Intervenciones Educativas en Profesionales Sanitarios.

El diamante del efecto combinado total cruza el eje vertical en el punto 24.02, con un Intervalo de Confianza del 95% de [23.91, 24.14]. Los resultados de los estudios analizados fueron altamente heterogéneos (I^2^ = 100%).

La prueba para el efecto global es altamente significativa (Z = 411.50, P < 0.00001).

#### Impacto Cuantitativo Sobre Conocimientos de Intervenciones en Competencias Culturales

Se analizaron 8 estudios, y el resultado global muestra una diferencia estandarizada de medias considerablemente alta, y un intervalo de confianza del 95%. El efecto total de la intervención sobre el conocimiento de competencias culturales fue estadísticamente significativo (P < 0.00001). Tal y como se muestra en la Figura 3. Siendo el estudio de Lin et al.^(24)^ el que aporta un mayor peso en relación con esta variable.

**Figura 3:** Mejora cuantitativa en conocimientos sobre competencias culturales tras Intervenciones Educativas en Profesionales Sanitarios.

#### Impacto Sobre la Confianza Profesional

Se evaluaron seis estudios para determinar la repercusión de factores relacionados con competencias culturales en la mejora de la confianza profesional. El análisis global refleja una diferencia estandarizada de medias significativa, con un intervalo de confianza del 95%, destacando un impacto positivo en la confianza. El efecto combinado de estas intervenciones resultó ser estadísticamente significativo con un valor de P < 0.00001. Estos resultados están presentados en la Figura 4. El estudio con mayor impacto en el análisis fue el de Oikarainen et al.^(31)^, que aporta el mayor peso respecto a esta variable específica.

**Figura 4:** Repercusión de factores relacionados con competencias culturales que influyen en la mejora de la confianza profesional.

### DISCUSIÓN

El presente análisis profundiza en el impacto de las intervenciones educativas en el desarrollo de la competencia cultural en el ámbito de la salud, revelando un énfasis predominante orientado en el desarrollo del entendimiento intercultural y las habilidades aplicadas al contexto clínico tal y como establecen Lin et al.^(24)^; Claramita et al.^(25)^ y otros autores^(26,27,28,29,30,31,32,35)^. La mayoría de los estudios analizados mostraron que las intervenciones influían de manera significativa en la mejora de la competencia cultural de estudiantes y enfermeras^(36)^.

Múltiples estudios concuerdan que la competencia cultural es un constructo multidimensional que abarca conocimientos, sensibilidad/conciencia, habilidades y, en algunos modelos, el deseo y el encuentro cultural. Las actividades educativas buscan desarrollar estas subáreas de manera integral y coordinada^(37,38)^. Como se indica en la Tabla 1, se puede apreciar las diversas acciones educativas utilizadas para fomentar un aprendizaje más profundo y transformador, reconociendo que un enfoque unidimensional resulta insuficiente para abordar la complejidad de este constructo^(39,40)^.

Las intervenciones educativas incluyen conferencias, sesiones de preguntas y respuestas, y debates para cubrir definiciones, importancia, teorías y el impacto cultural en la atención de salud. Sin embargo, se ha señalado que las charlas didácticas por sí solas pueden no ser suficientes para desarrollar todas las dimensiones de la competencia cultural, particularmente aquellas relacionadas con la sensibilidad cultural y las habilidades prácticas de aplicación en contextos clínicos reales^(36,37)^.

El uso de videos y películas, seguido de discusiones interactivas, representa una estrategia educativa efectiva que ayuda a comprender la diversidad cultural, eliminar estereotipos y prejuicios, y fomentar la reflexión sobre los propios valores y el impacto de los antecedentes culturales. Esta metodología permite a los participantes visualizar situaciones culturalmente complejas y desarrollar una mayor conciencia sobre las dinámicas interculturales en el ámbito sanitario^(40)^.

Los ejercicios de escritura reflexiva sobre la propia herencia cultural, a menudo combinados con dibujos y discusión, facilitan la autoexploración y la comprensión de cómo la cultura personal influye en las decisiones de salud. Se destaca que la reflexión es una parte crucial del diseño de actividades para la competencia cultural, ya que permite a los profesionales sanitarios reconocer sus propios sesgos culturales y desarrollar una mayor autoconciencia antes de trabajar con poblaciones diversas^(37)^.

En el desarrollo de estas intervenciones, se utilizan modelos reconocidos como el de Giger y Davidhizar, el Proceso de Competencia Cultural de Campinha-Bacote, y la Teoría del Cuidado Cultural (Sunrise Enabler) de Leininger. Estos modelos proporcionan marcos teóricos sólidos para la evaluación y el diseño de intervenciones culturalmente sensibles, ofreciendo estructuras conceptuales que guían tanto la implementación como la evaluación de los programas educativos^(37)^.

El énfasis en el “contexto clínico” sugiere que las intervenciones más efectivas no son solo teóricas, sino que incorporan casos prácticos, simulaciones o escenarios donde los estudiantes y profesionales deben aplicar sus conocimientos sobre diversidad cultural en situaciones reales de atención sanitaria. Entre estas estrategias destacan los estudios de caso interactivos, donde se presentan dilemas culturales y se discuten las mejores formas de abordarlos en un entorno clínico, las simulaciones con pacientes estandarizados que entrenan a los participantes a interactuar con “pacientes” de diferentes trasfondos culturales y a manejar situaciones delicadas, y las rotaciones clínicas o experiencias de inmersión cultural, siendo estas últimas una de las intervenciones más potentes que se incluyen en la literatura científica^(39,40)^.

Los estudios de caso y simulaciones permiten a los estudiantes analizar situaciones complejas, practicar habilidades en un entorno seguro y aplicar conocimientos a escenarios realistas. El juego de roles estructurado, en particular, puede ayudar a experimentar la desigualdad y reflexionar sobre los prejuicios, proporcionando una experiencia de aprendizaje transformadora que va más allá del conocimiento teórico hacia la comprensión vivencial de las dinámicas culturales en la atención sanitaria^(40)^.

La predominancia de mujeres en la población estudiada (71,5%) y una media de edad de los sujetos en las fases tempranas o intermedias de su trayectoria profesional, sugiere que el fomento de competencias culturales se está priorizando en etapas formativas fundamentales tal y como sugieren Álvarez-San Martín et al.^(34)^. Este enfoque podría resultar estratégico, considerando que la socialización temprana en competencias culturales podría tener un efecto duradero en la práctica profesional, al propiciar una base sólida para su desarrollo continuo.

La utilización de Facebook como herramienta educativa según Chang et al.^(26)^ y el empleo de los Círculos de Cultura Cavalcante^(33)^ reflejan una tendencia hacia la innovación pedagógica en la enseñanza de la competencia cultural. Giroux y Moreau^(41)^ en su artículo indican que las redes sociales desempeñan una función significativa en el desarrollo de las actividades académicas. En el caso de Facebook, su efectividad reside en su uso como una plataforma para discusiones en línea, intercambio de recursos multimedia y para fomentar la reflexión crítica sobre casos culturales fuera del aula, promoviendo la interacción continua y el aprendizaje colaborativo^(42,43)^.

Respecto a los Círculos de Cultura, este enfoque requiere de un método estructurado que implica diálogo, reflexión grupal y construcción colectiva de conocimiento sobre temas culturales. Es un enfoque que valora las experiencias vividas de los participantes y promueve la empatía y la comprensión mutua a través de la discusión guiada.

La integración de estas plataformas en el ámbito educativo presenta consideraciones relevantes para los marcos teóricos del aprendizaje y para el diseño instruccional de los programas académicos. Particularmente, surge la necesidad de conceptualizar y habilitar espacios virtuales que faciliten la conformación de comunidades de práctica y aprendizaje colaborativo entre los estudiantes, reconociendo el potencial pedagógico de estos entornos digitales para enriquecer la experiencia educativa y promover la construcción social del conocimiento^(42,43)^.

No obstante, se identifica una limitación metodológica significativa, y es la escasa utilización de grupos control, presentes en solo 8 de los 13 estudios analizados. Esta carencia sugiere la necesidad de estructuras metodológicas más robustas en futuras investigaciones para evaluar de manera concluyente la efectividad de estas intervenciones^(36,44)^.

La diversidad en las metodologías y herramientas de evaluación, junto con la variabilidad en las intervenciones, subraya la complejidad en la estandarización de las mediciones e intervenciones de competencia cultural tal y como han establecido otros autores como Kaiafas y Kennedy^(29)^ y Kula et al.^(30)^. Esta heterogeneidad plantea retos significativos para la estandarización y la comparación fiable de los hallazgos entre diferentes estudios^(36,45)^. A pesar de estas diferencias, la tendencia general hacia la mejora de la confianza y el conocimiento de las competencias culturales entre los participantes apunta a un efecto positivo de las intervenciones, afirmación con la que concuerdan Majda et al.^(32)^ y Zeidani et al.^(35)^.

La enfocada atención en la formación de estudiantes de enfermería refleja una vanguardia en esta área educativa, pero también marca la importancia de extender el alcance de tales estudios a otras especialidades sanitarias, para obtener una perspectiva más holística sobre la influencia y el impacto de las competencias culturales en el ámbito de la salud tal y como establece Oikarainen et al.^(31,40,45)^.

En el metaanálisis, se observa que todos los estudios individuales muestran un efecto positivo de las intervenciones (Figura 2). El tamaño del efecto combinado, indica una mejora significativa en las competencias culturales como resultado de las intervenciones. El valor de Z de 4.11 con un p-valor menor a 0.00001, indica una alta significación estadística del resultado global; en otras palabras, es muy poco probable que este resultado sea debido al azar. Si bien existe una considerable heterogeneidad entre los estudios —posiblemente debido a diferencias en las intervenciones, poblaciones estudiadas, o en cómo se midieron las competencias culturales—, la consistencia en la dirección del efecto refuerza la idea de que las intervenciones educativas tienen un impacto positivo en la mejora de las competencias culturales en profesionales sanitarios, lo que sugiere la relevancia de incorporar y potenciar estrategias de aprendizaje cultural en la formación continua de profesionales del ámbito sanitario^(36)^.

Truong et al.^(46)^ en su revisión de las revisiones indican que hay alguna evidencia de que las intervenciones para mejorar la competencia cultural pueden mejorar los resultados de salud del paciente/cliente, si bien la evidencia sobre resultados en pacientes sigue siendo limitada y heterogénea^(44)^.

Se evidenció que las intervenciones educativas tienen un efecto sustancial en el conocimiento de competencias culturales entre los profesionales sanitarios. El estudio de Lin et al.^(24)^ se destaca dentro del análisis, lo cual sugiere que la naturaleza o calidad de esta intervención en particular es especialmente efectiva^(24,36)^.

La robustez de los resultados refuerza la premisa del investigador de que la educación dirigida y bien estructurada puede ser un pilar en la mejora de la calidad del servicio en salud, lo que concuerda con lo propuesto por varios autores que realizaron revisiones similares en el pasado^(17,18,19,36,44)^.

Estos hallazgos evidencian que las intervenciones educativas incrementan la comprensión y desarrollan la capacidad de los profesionales para actuar con sensibilidad ante la diversidad cultural. Asimismo, los resultados subrayan la relevancia de considerar la especificidad metodológica de cada intervención, dado que ciertas estrategias formativas demuestran mayor efectividad que otras en el desarrollo de esta competencia fundamental para la atención sanitaria en contextos globalizados^(38,40,45)^.

En el análisis de los factores que influyen en la confianza profesional, se observa que las intervenciones de competencia cultural desempeñan un rol significativo, tal como se refleja en el estudio de Oikarainen et al.^(31)^, que emerge como el más influyente en este aspecto y en el de Osmancevic et al.^(36)^.

La investigación abarca una variedad de estudios que demuestran el impacto positivo de las intervenciones educativas en competencias culturales. La mejora en la confianza y el conocimiento cultural entre los profesionales de la salud señala que tales intervenciones son cruciales. Además, el enfoque en la formación de estudiantes de enfermería y el uso innovador de herramientas como Facebook sugieren una adaptación de los métodos de enseñanza a las tendencias actuales y las necesidades emergentes del sector sanitario^(40,42,43)^.

No obstante, este estudio presenta varias limitaciones que deben ser consideradas al interpretar los hallazgos. Como limitaciones del estudio se destaca la variabilidad en los tamaños muestrales y las características demográficas, lo que implica cautela en la generalización de resultados; además, aunque todos los estudios señalan una mejora, la alta heterogeneidad observada hace que la magnitud de dicha mejora varíe considerablemente.

Una de las limitaciones más evidentes es la ausencia de grupos control en una proporción considerable de los estudios, lo que dificulta la comparación objetiva de los resultados y la atribución causal de las mejoras a las intervenciones específicas. Además, la heterogeneidad observada en las metodologías y en las herramientas de evaluación utilizadas plantea retos para estandarizar y comparar los resultados de forma fiable tal como advierten Kaiafas y Kennedy^(29)^ y Kula et al.^(30)^. La variabilidad en los tamaños de muestra y las diferencias en las características demográficas de los participantes también limitan la capacidad de generalizar los hallazgos a la población en general. Finalmente, a pesar de que todos los estudios individuales apuntan a una mejora, la alta heterogeneidad en los efectos observados sugiere que la magnitud de esta mejora varía considerablemente, lo que exige cautela al inferir la magnitud del impacto global.

Para investigaciones futuras, sería beneficioso estructurar estudios con grupos control bien definidos y metodologías homogéneas para fortalecer la validez de las conclusiones. Asimismo, sería pertinente explorar el impacto de estas intervenciones educativas en una gama más amplia de disciplinas sanitarias y en diferentes etapas profesionales, no limitándose solo a la enfermería. Igualmente, sería valioso indagar sobre la permanencia a largo plazo de las competencias culturales adquiridas y cómo estas se aplican en la práctica diaria. Por último, se recomienda realizar ensayos controlados aleatorizados a gran escala para una evaluación más precisa de la efectividad de las intervenciones en competencia cultural y la adaptación contextualizada de programas educativos a diferentes y poblaciones de pacientes.

## CONCLUSIÓN

Las intervenciones educativas en competencia cultural son fundamentales para mejorar la calidad de la atención sanitaria, aumentando la satisfacción del paciente y las habilidades de comunicación intercultural de los profesionales. La competencia cultural es un proceso dinámico que requiere formación continua, auto-reflexión crítica y humildad cultural para superar sesgos personales.

Aunque existen desafíos significativos (barreras lingüísticas, diferencias culturales en concepciones de salud, condiciones laborales precarias), su superación es esencial mediante el desarrollo de habilidades comunicativas interculturales y la valoración del conocimiento local de las comunidades.

Persisten problemas para evaluar cambios actitudinales, las intervenciones cortas no afianzan el componente afectivo y el enfoque funcionalista domina sobre perspectivas críticas, mientras que precariedad laboral, influencias políticas y falta de recursos interrumpen la continuidad educativa.

En conjunto, la evidencia sugiere beneficios, pero se requieren estudios más sólidos que aclaren qué componentes pedagógicos generan mayores efectos y en qué contextos. Es prioritaria la estandarización de métricas más allá de la autoevaluación, incorporando resultados clínicos. Faltan ensayos aleatorizados y diseños longitudinales con seguimiento ≥6–12 meses para estimar el efecto y por ende la respuesta. Se necesitan análisis de moderadores y mediadores (perfil profesional, entorno asistencial, carga laboral, apoyo organizativo) para explicar heterogeneidad. Urge incorporar contextos infrarrepresentados (AP, emergencias, UCI; países de renta media-baja) y métodos mixtos que vinculen mecanismos con resultados. Finalmente, casi no existen evaluaciones económicas ni estudios sobre el impacto de intervenciones digitales.

## Data Availability

El conjunto de datos que respaldan las conclusiones de este estudio está disponible previa solicitud al autor correspondiente.
